# Atomic Force Microscopy Application for the Measurement of Infliximab Concentration in Healthy Donors and Pediatric Patients with Inflammatory Bowel Disease

**DOI:** 10.3390/jpm12060948

**Published:** 2022-06-10

**Authors:** Debora Curci, Marianna Lucafò, Pietro Parisse, Giuliana Decorti, Matteo Bramuzzo, Loredana Casalis, Gabriele Stocco

**Affiliations:** 1 Institute for Maternal and Child Health-IRCCS “Burlo Garofolo”, 34137 Trieste, Italy; debora.curci@burlo.trieste.it (D.C.); marianna.lucafo@burlo.trieste.it (M.L.); giuliana.decorti@burlo.trieste.it (G.D.); matteo.bramuzzo@burlo.trieste.it (M.B.); gabriele.stocco@burlo.trieste.it (G.S.); 2 Elettra Sincrotrone Trieste S.C.p.A., 34137 Trieste, Italy; loredana.casalis@elettra.eu; 3 Istituto Officina dei Materiali—CNR, 34149 Trieste, Italy; 4 Department of Medical, Surgical and Health Sciences, University of Trieste, 34127 Trieste, Italy

**Keywords:** atomic force microscopy, inflammatory bowel disease, infliximab, pediatric, nanoassay

## Abstract

The use of infliximab has completely changed the therapeutic landscape in inflammatory bowel disease. However, despite its proven efficacy to induce and maintain clinical remission, increasing evidence suggests that treatment failure may be associated with inadequate drug blood concentrations. The introduction of biosensors based on different nanostructured materials for the rapid quantification of drugs has been proposed for therapeutic drug monitoring. This study aimed to apply atomic force microscopy (AFM)-based nanoassay for the measurement of infliximab concentration in serum samples of healthy donors and pediatric IBD patients. This assay measured the height signal variation of a nanostructured gold surface covered with a self-assembled monolayer of alkanethiols. Inside this monolayer, we embedded the DNA conjugated with a tumor necrosis factor able to recognize the drug. The system was initially fine-tuned by testing known infliximab concentrations (0, 20, 30, 40, and 50 nM) in buffer and then spiking the same concentrations of infliximab into the sera of healthy donors, followed by testing pediatric IBD patients. A good correlation between height variation and drug concentration was found in the buffer in both healthy donors and pediatric IBD patients (*p*-value < 0.05), demonstrating the promising use of AFM nanoassay in TDM.

## 1. Introduction

Inflammatory bowel diseases (IBD) are complex disorders of the gastrointestinal tract, characterized by a relapsing and remitting course, and include Crohn’s disease (CD) and Ulcerative Colitis (UC). Patients with IBD must face long-term medical therapy and even surgical interventions [1]. Infliximab, an anti-TNF agent, represents a significant advance in the treatment of these chronic disorders thanks to its ability to induce and maintain clinical remission [2]. Dose optimization through therapeutic drug monitoring (TDM) is now a standard of care for therapy personalization, by which drug dosing is adjusted based on the stratification on immunogenic (presence of anti-drug antibodies), pharmacokinetic (insufficient drug trough concentrations without anti-drug antibodies), and pharmacodynamic (lack of efficacy despite adequate trough concentrations) loss of response (ECCO-ESPGHAN guideline) [3]. Even though several assays such as the radioimmunoassay, the homogeneous mobility shift assay, and the cell reporter gene assay–have been set up to measure the concentrations of infliximab and anti-drug antibodies, the enzyme-linked immunosorbent assay (ELISA) remains the most commonly used test for TDM in clinical practice [4,5,6]. Although these assays give reliable and reproducible results, they are expensive, especially when processing a limited number of samples [4]. Miniaturization is the key for the creation of point of care devices capable of testing low volumes of biological samples [7]. In recent years, by allowing the preparation of miniaturized devices and supports, nanotechnology offered the possibility of overcoming several limitations of the available assays with the possibility of measuring nanomolar protein concentrations in a small volume [8,9,10,11,12,13]. A variety of sensors based on different nanostructured materials and techniques have been developed in order to allow highly sensitive, non-invasive, and fast detection of biomarkers with the potential for being used as point of care devices [14]. Atomic force microscopy (AFM) has been used for realizing and analyzing miniaturized devices. Indeed, AFM is a scanning probe technique that combines a nanoscale resolution and a high imaging capacity; it is a valuable tool for the analysis of biological samples, with the possibility of performing measures in a liquid environment. Moreover, the capability of AFM to apply force with a nanometer-sized tip also allows the patterning of surfaces with high spatial resolution, favoring the creation of nanoarrays of molecules for biodetection purposes [15,16,17]. In particular, in recent years, the combination of AFM nanografting of single-stranded DNA (ssDNA) and DNA-directed immobilization (DDI) of conjugated binders allowed to confine high-affinity antigen-binding molecules on a surface and to create ligand-binding assays that are very sensitive, specific, with a relatively high throughput, that only requires small amounts of samples for the analysis [18,19]. Compared with the gold standard ELISA, multiplexing nanoarrays have several advantages: the high-throughput nature, efficiency in terms of time and cost, and the ability to measure one antigen in the context of multiple others; it has already been demonstrated as successful for the detection of cancer biomarkers [20,21].

Here, we applied the innovative AFM technique to measure infliximab serum concentrations in pediatric IBD patients.

## 2. Materials and Methods

### 2.1. AFM-Based Nanoassay

**Figure 1:**
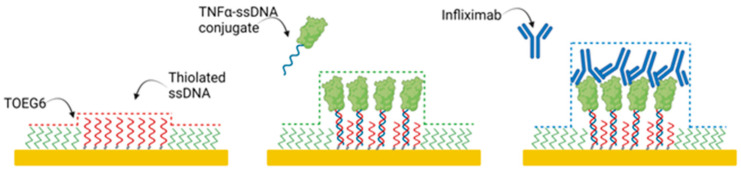
Infliximab measurement in an AFM-based nanoassay: a schematic representation of thiolated ssDNA patches fabricated through nanografting inside a TOEG6 carpet, TNFα-ssDNA conjugate binding, and infliximab detection. Image created with BioRender.com (accessed on 4 April 2022).

The AFM (XE-100 Park Instruments) based nanoassay measured the height variation of a nanostructured gold surface covered with a self-assembled monolayer (SAM) of alkanethiols. To avoid unspecific protein adsorption, alkanethiols were terminally functionalized with oligoethylene glycol (TOEG6). Inside this monolayer, we embedded proteins able to recognize the infliximab present in a solution, as represented in Figure 1.

In detail, single-stranded thiolated DNA (SH-cF9: SH-(CH2) 6-5′-CTTATCGCTTTATGACCGGACC-3′ from Biomers GmbH Ulm, Germany) patches are fabricated through nanografting inside a TOEG6-SAM carpet; the procedure has already been reported in the work of Ambrosetti and colleagues [20]. Ultraflat gold surfaces are coated with biorepellent self-assembled monolayers (TOEG6) through overnight incubation in a 300 μM TOEG6 in ethanol. The freshly prepared samples are glued into the AFM liquid cell using a Reprorubber to guarantee stability and chemical inertness. The cell is filled with TE buffer (Tris 10 mM, EDTA 1 mM, NaCl 1 M; pH 8) containing 5 μM of a thiolated ssDNA (SH-cF9). To fabricate the micrometer sized patches, we used NSC36 cantilevers (Mikromasch, spring constant = 0.6–2 N/m, radius of curvature < 10 nm) in contact mode with a scan rate of 1–2 Hz. The application of a force of roughly 100 nN allows the displacement of the TOEG6 molecules in favour of the thiolated ssDNA present in solution. The height of the ssDNA is then checked by topographic measurements in contact mode in the TE buffer (Tris 10 mM, EDTA 1 mM, NaCl 1 M; pH 8) using very soft cantilevers (CSC38 cantilevers, Mikromasch, spring constant = 0.003–0.006 N/m, radius of curvature <10 nm) with a contact force of 100 pN and a scan rate of 1 Hz. A new single ssDNA sequence complementary to the nanografted one (cF9-SH), conjugated with TNFα (cF9-TNFα conjugate), is immobilized on the nanospot via DNA–DNA interaction. This process is known as DNA-Directed Immobilization (DDI). To perform this operation, we incubated the gold chip with the ssDNA micrometer-sized patches with 100 nM of cF9-TNFα conjugate in the TE buffer for 2 h. The chip is then thoroughly washed with TE buffer to remove loosely bound conjugates and is imaged through topographic measurements in contact mode in the TE buffer using very soft cantilevers (CSC38 cantilevers, Mikromasch, spring constant = 0.003–0.006 N/m, radius of curvature <10 nm) with a contact force of 100 pN and a scan rate of 1 Hz. The final structures (the conjugate) composed by double-stranded DNA linked to TNFα on the top are now ready to recognize the analyte infliximab present in solution. The chip is therefore incubated with the infliximab sample for one hour, thoroughly washed with TE buffer, and imaged through topographic measurements in contact mode in TE buffer using very soft cantilevers (CSC38 cantilevers, Mikromasch, spring constant = 0.003–0.006 N/m, radius of curvature <10 nm) with a contact force of 100 pN and a scan rate of 1 Hz. The height variation on the patches before and after incubation with the sample is representative of the effective/not effective immobilization of the antibody on the sensing surface. 

To fine-tune the assay, several concentrations of infliximab (20, 30, 40, and 50 nM) were initially tested in the TE buffer. Afterwards, sera of six healthy donors spiked with known concentrations of infliximab below and within the therapeutic range (10, 20, 30, 40, and 50 nM, that correspond to 1.5, 3, 4.4, 6, and 7.5 µg/mL, respectively) were used to check the compatibility of the AFM system with serum sample and avoid unspecific measurements. In this preliminary study, we selected a concentration range between 0 and 7.5 µg/mL, which includes the therapeutic target (5 µg/mL during maintenance therapy) defined by the recent ECCO guideline to achieve the mucosal healing in Crohn’s disease [3]. 

The serum samples were diluted 1:100 in a physiologic solution with 10 mM EDTA added. The addition of EDTA, a chelator of Mg^2+^ ions, allows to maintain the integrity of the patches created during the nanografting phase, minimizing the action of nucleases, present in serum, on nanografted DNA. All the AFM images were taken at a frequency of 1 Hz and result from 256 × 256 lines. The images were recorded in gentle contact at minimum force values, and the topography analysis was determined using the Xei software, version 1.8.

### 2.2. Infliximab Patients’ Cohort

Patients with IBD treated with infliximab were enrolled at the Pediatric Gastroenterology Unit of the Institute for Maternal and Child Health IRCCS Burlo Garofolo, Trieste. Children were treated according to a therapeutic protocol consisting of intravenous administration of infliximab at 5 mg/kg in 2 h infusions at weeks 0, 2, 6 (induction phase), followed by a maintenance phase in which infusions were performed every 8 weeks. The inclusion criteria were age between 7 and 18 years, with previous diagnosis of IBD and treatment with infliximab. Infliximab was started as first line therapy or in case of treatment failure or intolerance to first-line therapies. The exclusion criteria were: patients with ileostomy or colostomy, disease needing surgery, or contemporary presence of other non-controlled pathologies that could affect the evaluation of the clinical score being not comparable to that of the other patients without this intervention. For each patient, clinical efficacy was assessed using then Pediatric Crohn’s Disease Activity Index (PCDAI) [22] and Pediatric Ulcerative Colitis Activity Index (PUCAI) [23] for CD and UC patients, respectively. The disease was considered in remission if indexes were < 10. Local Ethic Committee approval and appropriate informed consent were obtained from all patients’ parents or caregivers.

The sera of pediatric IBD patient were collected and analyzed by the gold standard ELISA (Progenika, Derio, Spain). The data obtained were then used to correlate infliximab concentration and height variation measured by AFM.

### 2.3. ELISA Assay

Infliximab levels were measured in the sera of six pediatric IBD patients using the commercial Promonitor ELISA assay (Progenika, Spain). The ELISA kit was used according to the manufacturers’ instructions, and the lower and upper limits of quantification were 0.017 and 14.4 μg/mL, respectively.

### 2.4. Statistical Analysis

Linear regression function was used to assess the correlation between infliximab concentration and signal variation (height variations). Graph-Pad Prism version 6.00 (GraphPad, La Jolla, CA, USA) was used for the one-way ANOVA statistical analysis.

## 3. Results

### 3.1. Measurement of Infliximab Concentration, in Buffer and Healthy Control Sera, by Atomic Force Microscopy

**Figure 2:**
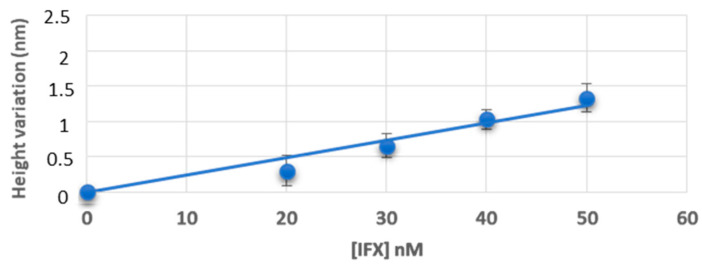
Calibration obtained in buffer. [IFX] = concentration of infliximab. Data are represented as mean ± standard deviation of three different measurements.

In Figure 2, we report the height variation of the patches after incubation of known concentrations of infliximab: a good correlation between height variation and drug concentration in buffer was found (linear regression: R^2^ = 0.97 and *p*-value = 0.003). 

**Figure 3:**
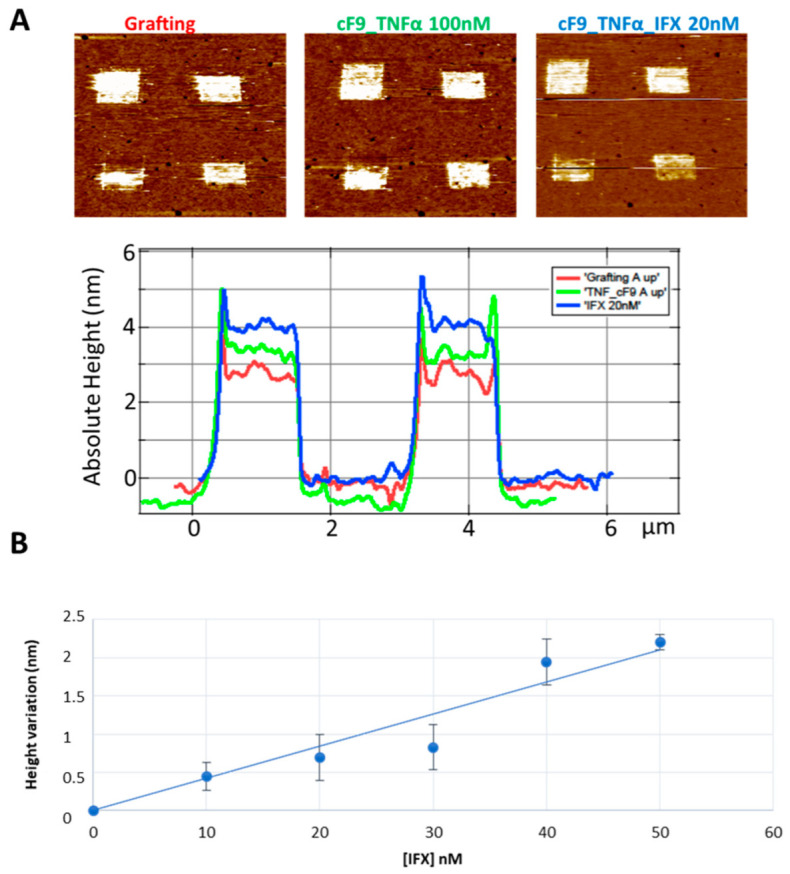
(**A**) An example of DNA nanostructures characterization by AFM topography: different DNA patches profiles with different height taken from AFM images (red line: ssDNA; green line: TNF-cF9 conjugate; blue line: infliximab (IFX) 20 nM); (**B**) the calibration line obtained in healthy donor sera spiked with IFX at different concentrations.

The same trend was also observed in the serum (Figure 3). We observe an increase of height after incubation with the TNFα-DNA conjugate and after the subsequent incubation with the serum containing infliximab (Figure 3A with results from serum spiked with infliximab 20 nM as representative image): the height variation also in this condition shows a good linear correlation with the drug concentration (linear regression: R^2^ = 0.93 and *p*-value = 0.002) (Figure 3B).

### 3.2. Measurement of Infliximab Concentration in Pediatric IBD Patients’ Sera, by Atomic Force Microscopy

The sera of six pediatric IBD patients treated with infliximab, whose serum concentration has been already determined by the Promonitor ELISA (Progenika, Spain) gold standard assay, were used for AFM-based nanoassay. The demographic characteristics of the IBD patients are summarized in Table 1.

Three pediatric patients (50%, median age = 13.13) were affected by CD and three by UC (50%, median age = 15.33). The 33.3% and 66.7% of CD and UC were, respectively, female. The disease activity index at inclusion was 14.2 (interquartile range, IQR: 7–19) and 15.1 (IQR: 8–21) for CD and UC, respectively. The infliximab quantifications obtained by ELISA are reported in Table 2 and permitted to include infliximab values below (0, 6.7 and 13.4 nM), above (65 nM), and in the therapeutic range (20 and 28 nM). 

**Figure 4:**
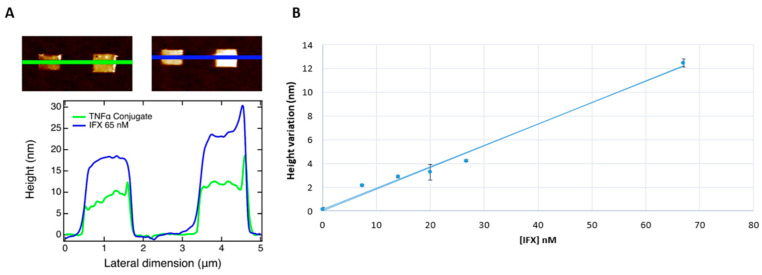
(**A**) Representative figure of DNA nanostructures characterization by AFM topography: different DNA patches profiles with different height taken from AFM images (green line: TNF-cF9 conjugate; blue line: IBD patient serum containing infliximab at a concentration of 65 nM); (**B**) calibration line obtained in six pediatric patients’ sera.

A good correlation between infliximab concentration measured by ELISA and height variation measured by AFM was assessed (linear regression: R^2^ = 0.98 and *p*-value = 0.025) (Figure 4). The pediatric IBD patient with 0 µg/mL infliximab level was positive for anti-drug antibodies, measured by ELISA assay. The presence of anti-drug antibodies did not affect the height measurement performed by AFM assay.

## 4. Discussion

In this study we used an AFM-based nanoassay to quantify infliximab in buffer TE, infliximab-spiked healthy donors, and infliximab-treated pediatric IBD patients’ sera. A good agreement was observed between signal variation and drug concentration both in buffer and in control sera. Considering the fact that the applied force, even if small, can reduce the observed height of biomolecules, and considering that IgG antibodies immobilised on a surface resulted to have heights comprised between 1 and 3.5 nm depending on their orientation [24], the height variations corresponding to the calibration in buffer and serum (Figure 2 and Figure 3) are compatible with the creation of a single layer of antibodies with different orientations.

The same trend was maintained in IBD patients’ sera, but a higher signal variation was observed compared to healthy donors. The increased height variation in the patients’ sera in comparison with the calibration curve may be due to clustering effects induced by other biomolecules such as anti-infliximab antibodies subtypes that do not affect the capability of the drug to recognize TNFα [25]. We argue that patients’ sera have different protein profiles with an increased expression of some proteins involved in inflammation, and these characteristics could affect the AFM analysis, inducing a clustering of molecules on the target patches increasing the height during TNFα-infliximab recognition. This consideration could be supported by Tomeková and colleagues who have shown qualitative and quantitative changes of proteins in blood serum samples of IBD patients compared to healthy donors by fluorescence analysis and AFM technique [26]. In particular, their morphological analysis of serum deposited on a specially cleaned slide (ultrasound, special nanopaper) revealed characteristic patterns of lipids, proteins, cholesterol, and other organic compounds with significant qualitative and quantitative differences in morphology (arrangement, size, particle distribution) between the samples of healthy individuals and IBD patients. 

Furthermore, AFM-based detection as well as other label-free approaches present as confounding factor the unspecific binding of other proteins/antibodies on the sensing area. This unspecific binding could be strongly reduced by the DDI approach and by the presence of antifouling TOEG6 carpet, and could be easily monitored in a multiplexing fashion by introducing control patches [18,20,21]. At the moment, the use of AFM for biomedical applications has been mainly limited for the analysis of stiffness of tissues and cells to help in diagnosing several diseases (neurodegenerative diseases, cancer, cardiovascular diseases, etc.), but also for detecting specific biomolecules [27]. Nonetheless, the reported nano-assay approach is quite unique, although similar attempts building on the seminal work of Sahin [28] have been made, mainly for miRNA analysis and DNA hybridization.

## 5. Conclusions

In conclusion, we successfully demonstrated the promising use of AFM nanoassay as a potential sensitive tool for TDM for pediatric IBD patients in treatment with infliximab. This preliminary study has some limitations regarding the small sample size and the higher signal variation observed in IBD patients’ sera. Thus, further AFM analysis including higher concentrations of infliximab and modifications in the technique will be considered to avoid, among other aspects, the detection of particles present in the serum of patients, in view of its possible application in clinical practice.

## Figures and Tables

**Table 1:** Demographic and clinical characteristics of the IBD population enrolled.

	Overall (*n* = 6)	Crohn’s Disease (*n* = 3)	Ulcerative Colitis (*n* = 3)
Age-years (IQR)	14.88 (12.7–16.1)	13.13 (10.8–15.7)	15.33 (13.1–16.1)
Disease duration—months (IQR)	39.4 (25.2–51.6)	43.18 (28.4–56.6)	22.6 (17.3–29.5)
Disease activity index at inclusion			
PCDAI (IQR)		14.2 (7–19)	-
PUCAI (IQR)		-	15.1 (8–21.3)
Paris Classification:			
Localization E3/E4 (%)	-	0	3 (100)
Localization L3/L4 (%)	-	3 (100)	0
Behavior B1 (%)	-	3 (100)	3 (100)
Maintenance treatment	6	3	3
Other biologics before infliximab (yes/no)	no	no	no
Gender			
Female (%)	3 (50)	1 (33.3)	2 (66.7)
Male (%)	3 (50)	2 (66.7)	1 (33.3)

Abbreviations: IQR, interquartile range, PCDAI, Pediatric Crohn’s disease activity index; PUCAI, Ulcerative Colitis activity index.

**Table 2:** ELISA measurements in the pediatric IBD cohort.

Pediatric IBD Patient	Infliximab (nM)	Infliximab (µg/mL)
1	0	0.0
2	6.7	1.0
3	13.4	2.0
4	20	3.0
5	28	4.2
6	65	9.75

Abbreviations: IBD, inflammatory bowel disease.

## Data Availability

The raw data supporting the conclusions of this article will be made available by the authors, without undue reservation.

## References

[1] Berg D.R., Colombel J.-F., Ungaro R. (2019). The Role of Early Biologic Therapy in Inflammatory Bowel Disease. Inflamm. Bowel Dis..

[2] Papamichael K., Lin S., Moore M., Papaioannou G., Sattler L., Cheifetz A.S. (2019). Infliximab in inflammatory bowel disease. Ther. Adv. Chronic Dis..

[3] Van Rheenen P.F., Aloi M., Assa A., Bronsky J., Escher J.C., Fagerberg U.L., Gasparetto M., Gerasimidis K., Griffiths A., Henderson P. (2021). The Medical Management of Paediatric Crohn’s Disease: An ECCO-ESPGHAN Guideline Update. J. Crohn’s Coliti.

[4] Franca R., Curci D., Lucafò M., Decorti G., Stocco G. (2019). Therapeutic drug monitoring to improve outcome of anti-TNF drugs in pediatric inflammatory bowel disease. Expert Opin. Drug Metab. Toxicol..

[5] Nasser Y., Labetoulle R., Harzallah I., Berger A.-E., Roblin X., Paul S. (2018). Comparison of Point-of-Care and Classical Immunoassays for the Monitoring Infliximab and Antibodies Against Infliximab in IBD. Am. J. Dig. Dis..

[6] Casteele N.V. (2016). Assays for measurement of TNF antagonists in practice. Front. Gastroenterol..

[7] Tüdős A.J., Besselink G.A.J., Schasfoort R.B.M. (2001). Trends in miniaturized total analysis systems for point-of-care testing in clinical chemistry. Lab Chip.

[8] Huang S., Wang W., Li J., Zhang T., Liang Y., Wang Q., Jiang Z. (2021). Multifunctional DNA mediated spatially confined assembly for antibody orientation: Surpassing sensitivity and accuracy for rituximab detection. Chem. Eng. J..

[9] Imaeda H., Andoh A., Fujiyama Y. (2011). Development of a new immunoassay for the accurate determination of anti-infliximab antibodies in inflammatory bowel disease. J. Gastroenterol..

[10] Lu J., Spasic D., Delport F., Van Stappen T., Detrez I., Daems D., Vermeire S., Gils A., Lammertyn J. (2017). Immunoassay for Detection of Infliximab in Whole Blood Using a Fiber-Optic Surface Plasmon Resonance Biosensor. Anal. Chem..

[11] Muneer S., Ayoko G.A., Islam N., Izake E.L. (2019). Utilizing the thiol chemistry of biomolecules for the rapid determination of anti-TNF-α drug in blood. Talanta.

[12] Thoren K.L., Pasi B., Delgado J.C., Wu A.H., Lynch K.L. (2018). Quantitation of Infliximab and Detection of Antidrug Antibodies in Serum by Use of Surface Plasmon Resonance. J. Appl. Lab. Med..

[13] Zeni L., Perri C., Cennamo N., Arcadio F., D’Agostino G., Salmona M., Beeg M., Gobbi M. (2020). A portable optical-fibre-based surface plasmon resonance biosensor for the detection of therapeutic antibodies in human serum. Sci. Rep..

[14] Zhang Y., Chan H.F., Leong K.W. (2012). Advanced materials and processing for drug delivery: The past and the future. Adv. Drug Deliv. Rev..

[15] Alsteens D., Gaub H.E., Newton R., Pfreundschuh M., Gerber C., Muller D.J. (2017). Atomic force microscopy-based characterization and design of biointerfaces. Nat. Rev. Mater..

[16] Menotta M., Biagiotti S., Streppa L., Rossi L., Magnani M. (2015). Label-free quantification of Tacrolimus in biological samples by atomic force microscopy. Anal. Chim. Acta.

[17] Steffens C., Leite F.L., Bueno C.C., Manzoli A., Herrmann P.S.D.P. (2012). Atomic Force Microscopy as a Tool Applied to Nano/Biosensors. Sensors.

[18] Bano F., Fruk L., Sanavio B., Glettenberg M., Casalis L., Niemeyer C.M., Scoles G. (2009). Toward Multiprotein Nanoarrays Using Nanografting and DNA Directed Immobilization of Proteins. Nano Lett..

[19] Castronovo M., Scaini D. (2011). The Atomic Force Microscopy as a Lithographic Tool: Nanografting of DNA Nanostructures for Biosensing Applications. Methods Mol. Biol..

[20] Ambrosetti E., Paoletti P., Bosco A., Parisse P., Scaini D., Tagliabue E., De Marco A., Casalis L. (2017). Quantification of Circulating Cancer Biomarkers via Sensitive Topographic Measurements on Single Binder Nanoarrays. ACS Omega.

[21] Ganau M., Bosco A., Palma A., Corvaglia S., Parisse P., Fruk L., Beltrami A.P., Cesselli D., Casalis L., Scoles G. (2015). A DNA-based nano-immunoassay for the label-free detection of glial fibrillary acidic protein in multicell lysates. Nanomed. Nanotechnol. Biol. Med..

[22] Hyams J.S., Ferry G.D., Mandel F.S., Gryboski J.D., Kibort P.M., Kirschner B.S., Griffiths A.M., Katz A.J., Grand R.J., Boyle J.T. (1991). Development and validation of a pediatric Crohn’s disease activity index. J. Pediatric Gastroenterol. Nutr..

[23] Turner D., Otley A.R., Mack D., Hyams J., de Bruijne J., Uusoue K., Walters T.D., Zachos M., Mamula P., Beaton D.E. (2007). Development, Validation, and Evaluation of a Pediatric Ulcerative Colitis Activity Index: A Prospective Multicenter Study. Gastroenterology.

[24] Vilhena J.G., Dumitru A.C., Herruzo E.T., Mendieta-Moreno J.I., Garcia R., Serena P.A., Pérez R. (2016). Adsorption orientations and immunological recognition of antibodies on graphene. Nanoscale.

[25] Bendtzen K. (2015). Immunogenicity of Anti-TNF-α Biotherapies: II. Clinical Relevance of Methods Used for Anti-Drug Antibody Detection. Front. Immunol..

[26] Tomečková V., Tóth Š., Tóth T., Komanický V., Krajčíková K., Široká M., Glinská G., Pella D., Mašlanková J., Tomečko M. (2018). Analysis of Bowel Diseases from Blood Serum by Autofluorescence and Atomic Force Microscopy Techniques. Open Chem..

[27] Kwon T., Gunasekaran S., Eom K. (2019). Atomic force microscopy-based cancer diagnosis by detecting cancer-specific biomolecules and cells. Biochim. Biophys. Acta.

[28] Husale S., Persson H.H.J., Sahin O. (2009). DNA nanomechanics allows direct digital detection of complementary DNA and microRNA targets. Nature.

